# (5*E*)-5-(4-Hydr­oxy-3-methoxy­benzyl­idene)-2-thioxo-1,3-thia­zolidin-4-one methanol monosolvate

**DOI:** 10.1107/S1600536809039567

**Published:** 2009-10-03

**Authors:** Durre Shahwar, M. Nawaz Tahir, Muhammad Asam Raza, Maria Saddaf, Sana Majeed

**Affiliations:** a Department of Chemistry, Government College University, Lahore, Pakistan; b Department of Physics, University of Sargodha, Sargodha, Pakistan

## Abstract

In the title compound, C_11_H_9_NO_3_S_2_·CH_4_O, the dihedral angle between the aromatic rings is 3.57 (16)° and intra­molecular O—H⋯O and C—H⋯S inter­actions occur. In the crystal, the thia­zolidin-4-one mol­ecules are linked by N—H⋯O hydrogen bonds, forming chains. The hydrogen-bond motifs lead to *S*(5), *S*(6) and *R*_3_^3^(8) ring motifs. There exist C=O⋯π inter­actions between the heterocyclic rings and π–π inter­actions between the heterocyclic and benzene rings at distances of 3.455 (2) and 3.602 (2) Å, respectively. The methanol solvent mol­ecule is disordered over two sets of sites in a 0.542 (9):0.458 (9) ratio.

## Related literature

For related structures, see: Barreiro *et al.* (2007 ▶); Shahwar *et al.* (2009 ▶). For graph-set notation, see: Bernstein *et al.* (1995 ▶).
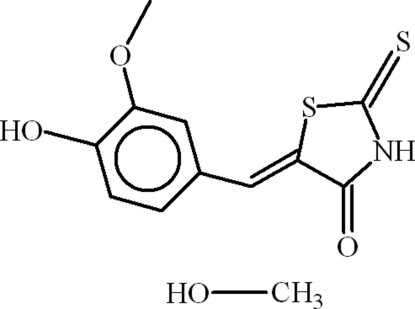


## Experimental

### 

#### Crystal data

C_11_H_9_NO_3_S_2_·CH_4_O
*M* *_r_* = 299.35
Orthorhombic, 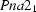

*a* = 17.731 (2) Å
*b* = 11.7528 (14) Å
*c* = 6.5715 (6) Å
*V* = 1369.4 (3) Å^3^
*Z* = 4
Mo *K*α radiation
μ = 0.40 mm^−1^
*T* = 296 K
0.26 × 0.13 × 0.12 mm

#### Data collection

Bruker Kappa APEXII CCD diffractometer
Absorption correction: multi-scan (*SADABS*; Bruker, 2007 ▶) *T*_min_ = 0.942, *T*_max_ = 0.955
7574 measured reflections
2472 independent reflections
1807 reflections with *I* > 2σ(*I*)
*R*_int_ = 0.045

#### Refinement

*R*[*F* ^2^ > 2σ(*F* ^2^)] = 0.039
*wR*(*F* ^2^) = 0.070
*S* = 1.02
2472 reflections
185 parameters
1 restraint
H atoms treated by a mixture of independent and constrained refinement
Δρ_max_ = 0.17 e Å^−3^
Δρ_min_ = −0.21 e Å^−3^
Absolute structure: Flack (1983 ▶), 829 Friedal Pairs
Flack parameter: 0.01 (8)

### 

Data collection: *APEX2* (Bruker, 2007 ▶); cell refinement: *SAINT* (Bruker, 2007 ▶); data reduction: *SAINT*; program(s) used to solve structure: *SHELXS97* (Sheldrick, 2008 ▶); program(s) used to refine structure: *SHELXL97* (Sheldrick, 2008 ▶); molecular graphics: *ORTEP-3* (Farrugia, 1997 ▶) and *PLATON* (Spek, 2009 ▶); software used to prepare material for publication: *WinGX* (Farrugia, 1999 ▶) and *PLATON*.

## Supplementary Material

Crystal structure: contains datablocks global, I. DOI: 10.1107/S1600536809039567/hb5118sup1.cif

Structure factors: contains datablocks I. DOI: 10.1107/S1600536809039567/hb5118Isup2.hkl

Additional supplementary materials:  crystallographic information; 3D view; checkCIF report

## Figures and Tables

**Table 1:** Hydrogen-bond geometry (Å, °)

*D*—H⋯*A*	*D*—H	H⋯*A*	*D*⋯*A*	*D*—H⋯*A*
O1—H1⋯O2	0.88 (3)	2.21 (3)	2.641 (3)	109 (3)
C2—H2⋯S1	0.93	2.66	3.349 (3)	132
O1—H1⋯O4*A*^i^	0.88 (3)	1.85 (3)	2.622 (7)	145 (3)
N1—H1*N*⋯O1^ii^	0.86	2.05	2.899 (3)	169
O4*A*—H4*A*⋯O3^iii^	0.96 (8)	1.79 (8)	2.744 (7)	173 (7)
C12*A*—H12*A*⋯O3^iv^	0.96	2.37	3.150 (5)	139

Symmetry codes: (i) 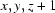; (ii) 
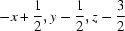
; (iii) 
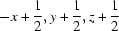
; (iv) 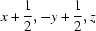. *Cg*2 is the centroid of the C1–C6 benzene ring.

**Fig. 1.**
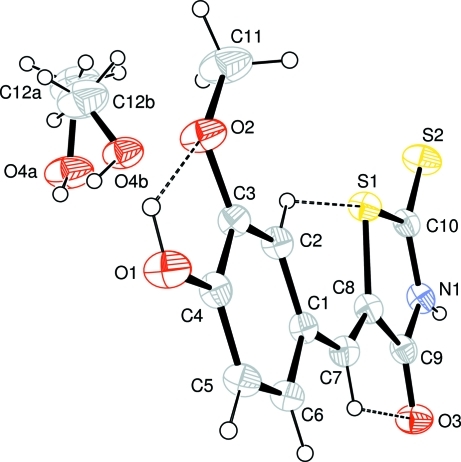
View of (I) with displacement ellipsoids drawn at the 50% probability level. H-atoms are shown by small circles of arbitrary radius. The dotted line represent the intramolecular H-bond.

**Fig. 2.**
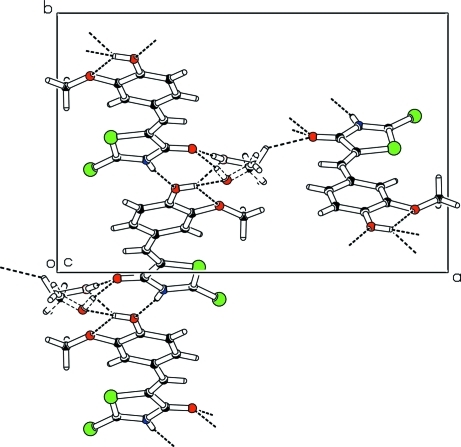
The partial packing of (I), which shows that molecules form polymeric chains.

## supplementary crystallographic information

### Comment

We have recently reported the crystal structure of (5*Z*)-5-(2-Hydroxybenzylidene)-2-thioxo-1,3-thiazolidin-4-one - methanol (1:0.5) (Shahwar *et al.*, 2009). In continuation of synthesizing various derivatives of rhodanine, the title compound (I, Fig. 1), is being reported.

The crystal structure of (II) 5-(4-Hydroxybenzylidene)-2-thioxo-1,3-thiazolidin-4-one dimethylsulfoxide solvate (Barreiro, *et al.*, 2007) has been published. The title compound (I) differs from (II) due to attachment of methoxy group adjacent to the hydroxy group and due to solvate i.e methanol instead of dimethylsulfoxide.

In the title molecule there exist interamolecular H-bondings of O—H···O, C—H···O and S—H···O types (Table 1, Fig. 1) forming two S(5) and one S(6) ring motif (Bernstein *et al.*, 1995). The role of disordered methanol solvate is to interlink the molecules through O—H···O type of H-bondings forming *R*_3_^3^(8) ring motifs (Fig. 2). The molecules are stabilized in the form of infinite one dimensional polymeric chains. There exist π–π interactions between the centroids of heterocyclic ring *Cg*1 (C8/C9/N1/C10/S1) and the benzene ring *Cg*2 (C1—C6). The distance between the centroids *Cg*1 →*Cg*2 is 3.455 (2) Å due to symmetry (*x*, *y*, ∓1 + *z*) and for *Cg*2 →*Cg*1 is 3.602 (2) Å due to symmetry (1/2 - *x*, ∓1/2 + *y*, ∓1/2 + *z*), respectively. The molecules may also be stabilized due C==O···π interaction (Table 1). The methanol molecule is disordered over two sites with an occupancy ratio of 0.542 (9):0.458 (9).

### Experimental

Rhodanine (0.266 g, 0.2 mol), 4-hydroxy-3-methoxybenzaldehyde (0.304 g, 0.2 mol) and K_2_CO_3_ (0.553 g, 0.4 mol) were dissolved in 10 ml distilled water at room temperature. The stirring was continued for 24 h and reaction was monitored by TLC. The precipitates were formed during neutalization of the reaction mixture with 5% HCl. The precipitates were filtered off and washed with saturated solution of NaCl. The crude material obtained was recrystalized in methanol to affoard dark brown needles of (I).

### Refinement

The coordinates of H1 and H4A attached with O1 and O4A respectively, were refined.

The H-atoms were positioned geometrically with O–H = 0.82, N–H = 0.86, C–H = 0.93 and 0.96 Å for aromatic like and methyl H atoms and constrained to ride on their parent atoms, with *U*_iso_(H) = xUeq(C, N, O), where *x* = 1.5 for methyl and *x* = 1.2 for all other H atoms.

### Crystal data

**Table:**

C_11_H_9_NO_3_S_2_·CH_4_O	*F*(000) = 624
*M**_r_* = 299.35	*D*_x_ = 1.452 Mg m^−^^3^
Orthorhombic, *P**n**a*2_1_	Mo *K*α radiation, λ = 0.71073 Å
Hall symbol: P 2c -2n	Cell parameters from 2472 reflections
*a* = 17.731 (2) Å	θ = 2.9–27.1°
*b* = 11.7528 (14) Å	µ = 0.40 mm^−^^1^
*c* = 6.5715 (6) Å	*T* = 296 K
*V* = 1369.4 (3) Å^3^	Cut needle, dark brown
*Z* = 4	0.26 × 0.13 × 0.12 mm

### Data collection

**Table:**

Bruker Kappa APEXII CCD diffractometer	2472 independent reflections
Radiation source: fine-focus sealed tube	1807 reflections with *I* > 2σ(*I*)
graphite	*R*_int_ = 0.045
Detector resolution: 7.50 pixels mm^-1^	θ_max_ = 27.1°, θ_min_ = 2.9°
ω scans	*h* = −22→22
Absorption correction: multi-scan (*SADABS*; Bruker, 2007)	*k* = −13→15
*T*_min_ = 0.942, *T*_max_ = 0.955	*l* = −5→8
7574 measured reflections	

### Refinement

**Table:**

Refinement on *F*^2^	Secondary atom site location: difference Fourier map
Least-squares matrix: full	Hydrogen site location: inferred from neighbouring sites
*R*[*F*^2^ > 2σ(*F*^2^)] = 0.039	H atoms treated by a mixture of independent and constrained refinement
*wR*(*F*^2^) = 0.070	*w* = 1/[σ^2^(*F*_o_^2^) + (0.0145*P*)^2^ + 0.2762*P*] where *P* = (*F*_o_^2^ + 2*F*_c_^2^)/3
*S* = 1.02	(Δ/σ)_max_ < 0.001
2472 reflections	Δρ_max_ = 0.17 e Å^−^^3^
185 parameters	Δρ_min_ = −0.21 e Å^−^^3^
1 restraint	Absolute structure: Flack (1983), 829 Friedal Pairs
Primary atom site location: structure-invariant direct methods	Flack parameter: 0.01 (8)

### Special details

**Table:**

Geometry. Bond distances, angles *etc*. have been calculated using the rounded fractional coordinates. All su's are estimated from the variances of the (full) variance-covariance matrix. The cell e.s.d.'s are taken into account in the estimation of distances, angles and torsion angles
Refinement. Refinement of *F*^2^ against ALL reflections. The weighted *R*-factor *wR* and goodness of fit *S* are based on *F*^2^, conventional *R*-factors *R* are based on *F*, with *F* set to zero for negative *F*^2^. The threshold expression of *F*^2^ > σ(*F*^2^) is used only for calculating *R*-factors(gt) *etc*. and is not relevant to the choice of reflections for refinement. *R*-factors based on *F*^2^ are statistically about twice as large as those based on *F*, and *R*- factors based on ALL data will be even larger.

### Fractional atomic coordinates and isotropic or equivalent isotropic displacement parameters (Å^2^)

**Table:**

	*x*	*y*	*z*	*U*_iso_*/*U*_eq_	Occ. (<1)
S1	0.36194 (4)	0.02090 (7)	0.57918 (14)	0.0440 (3)	
S2	0.41466 (5)	−0.10304 (9)	0.20833 (15)	0.0609 (4)	
O1	0.30380 (12)	0.3234 (2)	1.4811 (3)	0.0514 (9)	
O2	0.41511 (11)	0.2575 (2)	1.2416 (3)	0.0540 (9)	
O3	0.15476 (12)	−0.0232 (2)	0.4387 (3)	0.0531 (9)	
N1	0.27209 (14)	−0.0654 (2)	0.3174 (4)	0.0403 (9)	
C1	0.25139 (18)	0.1479 (3)	0.9536 (4)	0.0352 (10)	
C2	0.32661 (18)	0.1714 (3)	1.0013 (5)	0.0383 (11)	
C3	0.34462 (16)	0.2296 (3)	1.1767 (5)	0.0377 (11)	
C4	0.28684 (17)	0.2662 (3)	1.3082 (4)	0.0373 (11)	
C5	0.21274 (17)	0.2455 (3)	1.2609 (5)	0.0412 (11)	
C6	0.19503 (16)	0.1858 (3)	1.0864 (5)	0.0411 (11)	
C7	0.22722 (18)	0.0873 (3)	0.7745 (5)	0.0410 (11)	
C8	0.26494 (16)	0.0363 (3)	0.6206 (4)	0.0376 (11)	
C9	0.22317 (17)	−0.0188 (3)	0.4558 (5)	0.0405 (11)	
C10	0.34766 (17)	−0.0547 (3)	0.3526 (5)	0.0392 (11)	
C11	0.47733 (18)	0.2154 (4)	1.1321 (6)	0.0780 (18)	
O4A	0.4252 (3)	0.4356 (7)	0.5888 (10)	0.078 (3)	0.542 (9)
C12A	0.5055 (2)	0.4123 (5)	0.6066 (13)	0.096 (2)	0.542 (9)
O4B	0.4343 (2)	0.3568 (4)	0.6677 (9)	0.059 (3)	0.458 (9)
C12B	0.4913 (2)	0.4044 (4)	0.6141 (9)	0.096 (2)	0.458 (9)
H1	0.3525 (17)	0.334 (3)	1.499 (5)	0.0617*	
H1N	0.25564	−0.10029	0.21132	0.0483*	
H2	0.36478	0.14769	0.91393	0.0460*	
H5	0.17457	0.27152	1.34604	0.0495*	
H6	0.14477	0.17047	1.05653	0.0492*	
H7	0.17507	0.08219	0.76231	0.0494*	
H11A	0.47613	0.13375	1.13217	0.1166*	
H11B	0.52316	0.24114	1.19480	0.1166*	
H11C	0.47516	0.24266	0.99447	0.1166*	
H4A	0.401 (4)	0.449 (6)	0.717 (13)	0.0935*	0.542 (9)
H12C	0.51734	0.39385	0.74524	0.1441*	0.542 (9)
H12A	0.53358	0.47831	0.56588	0.1441*	0.542 (9)
H12B	0.51849	0.34937	0.52036	0.1441*	0.542 (9)
H4B	0.40848	0.40061	0.73547	0.0709*	0.458 (9)
H12D	0.50918	0.45230	0.72210	0.1441*	0.458 (9)
H12E	0.48086	0.44993	0.49615	0.1441*	0.458 (9)
H12F	0.52902	0.34877	0.58154	0.1441*	0.458 (9)

### Atomic displacement parameters (Å^2^)

**Table:**

	*U*^11^	*U*^22^	*U*^33^	*U*^12^	*U*^13^	*U*^23^
S1	0.0445 (4)	0.0523 (5)	0.0353 (4)	0.0038 (4)	−0.0056 (4)	−0.0094 (5)
S2	0.0552 (5)	0.0804 (8)	0.0472 (5)	0.0164 (5)	0.0008 (5)	−0.0137 (6)
O1	0.0444 (14)	0.0726 (19)	0.0372 (13)	0.0026 (13)	0.0019 (11)	−0.0231 (12)
O2	0.0383 (13)	0.0796 (17)	0.0442 (14)	−0.0046 (12)	0.0060 (11)	−0.0230 (13)
O3	0.0435 (13)	0.0691 (18)	0.0467 (14)	0.0004 (12)	−0.0084 (11)	−0.0146 (13)
N1	0.0527 (17)	0.0388 (18)	0.0293 (14)	0.0048 (14)	−0.0049 (12)	−0.0101 (13)
C1	0.0417 (17)	0.0343 (19)	0.0295 (17)	0.0022 (14)	0.0031 (14)	−0.0013 (14)
C2	0.0442 (19)	0.041 (2)	0.0297 (17)	0.0029 (16)	0.0060 (14)	−0.0062 (16)
C3	0.0389 (17)	0.040 (2)	0.0341 (19)	0.0002 (15)	0.0035 (15)	−0.0030 (17)
C4	0.047 (2)	0.037 (2)	0.0278 (18)	0.0023 (15)	0.0007 (15)	−0.0093 (16)
C5	0.0414 (19)	0.051 (2)	0.0313 (18)	0.0070 (17)	0.0024 (15)	−0.0069 (16)
C6	0.0372 (17)	0.045 (2)	0.0410 (18)	0.0037 (14)	−0.0008 (18)	−0.001 (2)
C7	0.0437 (18)	0.041 (2)	0.0383 (19)	0.0002 (16)	−0.0016 (15)	0.0005 (17)
C8	0.0464 (17)	0.039 (2)	0.0275 (19)	0.0024 (14)	−0.0081 (14)	−0.0034 (15)
C9	0.048 (2)	0.037 (2)	0.0364 (18)	0.0040 (17)	−0.0041 (16)	−0.0005 (16)
C10	0.047 (2)	0.038 (2)	0.0327 (18)	0.0049 (15)	−0.0088 (15)	−0.0018 (15)
C11	0.044 (2)	0.113 (4)	0.077 (3)	−0.005 (2)	0.0177 (18)	−0.026 (3)
O4A	0.048 (3)	0.119 (6)	0.067 (4)	0.004 (3)	0.005 (3)	−0.039 (5)
C12A	0.064 (3)	0.131 (5)	0.093 (4)	−0.008 (3)	0.011 (3)	−0.019 (4)
O4B	0.043 (3)	0.079 (6)	0.055 (4)	−0.001 (3)	0.006 (3)	−0.020 (4)
C12B	0.064 (3)	0.131 (5)	0.093 (4)	−0.008 (3)	0.011 (3)	−0.019 (4)

### Geometric parameters (Å, °)

**Table:**

S1—C8	1.751 (3)	C3—C4	1.408 (4)
S1—C10	1.752 (3)	C4—C5	1.372 (4)
S2—C10	1.623 (3)	C5—C6	1.381 (5)
O1—C4	1.354 (4)	C7—C8	1.353 (4)
O2—C3	1.361 (4)	C8—C9	1.463 (4)
O2—C11	1.407 (4)	C2—H2	0.9300
O3—C9	1.219 (4)	C5—H5	0.9300
O1—H1	0.88 (3)	C6—H6	0.9300
O4A—C12A	1.455 (7)	C7—H7	0.9300
O4B—C12B	1.208 (6)	C11—H11A	0.9600
O4A—H4A	0.96 (8)	C11—H11B	0.9600
O4B—H4B	0.8200	C11—H11C	0.9600
N1—C10	1.366 (4)	C12A—H12B	0.9600
N1—C9	1.371 (4)	C12A—H12C	0.9600
N1—H1N	0.8600	C12A—H12A	0.9600
C1—C2	1.398 (5)	C12B—H12D	0.9600
C1—C7	1.441 (4)	C12B—H12E	0.9600
C1—C6	1.400 (4)	C12B—H12F	0.9600
C2—C3	1.378 (5)		
			
C8—S1—C10	92.44 (14)	S2—C10—N1	126.0 (3)
C3—O2—C11	118.4 (3)	S1—C10—S2	124.63 (19)
C4—O1—H1	114 (2)	C3—C2—H2	120.00
C12A—O4A—H4A	114 (4)	C1—C2—H2	120.00
C12B—O4B—H4B	110.00	C4—C5—H5	120.00
C9—N1—C10	118.1 (3)	C6—C5—H5	120.00
C10—N1—H1N	121.00	C5—C6—H6	119.00
C9—N1—H1N	121.00	C1—C6—H6	119.00
C2—C1—C7	124.4 (3)	C8—C7—H7	113.00
C2—C1—C6	118.6 (3)	C1—C7—H7	113.00
C6—C1—C7	117.0 (3)	O2—C11—H11C	109.00
C1—C2—C3	120.5 (3)	O2—C11—H11A	110.00
O2—C3—C4	113.7 (3)	O2—C11—H11B	109.00
O2—C3—C2	126.5 (3)	H11B—C11—H11C	109.00
C2—C3—C4	119.8 (3)	H11A—C11—H11B	109.00
O1—C4—C5	119.4 (3)	H11A—C11—H11C	109.00
C3—C4—C5	120.3 (3)	O4A—C12A—H12B	109.00
O1—C4—C3	120.4 (3)	O4A—C12A—H12C	109.00
C4—C5—C6	119.8 (3)	O4A—C12A—H12A	109.00
C1—C6—C5	121.2 (3)	H12A—C12A—H12C	109.00
C1—C7—C8	133.1 (3)	H12B—C12A—H12C	110.00
S1—C8—C9	109.7 (2)	H12A—C12A—H12B	109.00
S1—C8—C7	130.4 (2)	O4B—C12B—H12D	109.00
C7—C8—C9	120.0 (3)	O4B—C12B—H12E	109.00
O3—C9—C8	126.2 (3)	O4B—C12B—H12F	109.00
N1—C9—C8	110.3 (3)	H12D—C12B—H12E	110.00
O3—C9—N1	123.5 (3)	H12D—C12B—H12F	110.00
S1—C10—N1	109.4 (2)	H12E—C12B—H12F	110.00
			
C10—S1—C8—C7	179.4 (4)	C1—C2—C3—O2	179.7 (3)
C10—S1—C8—C9	0.3 (3)	C1—C2—C3—C4	−0.4 (5)
C8—S1—C10—S2	179.6 (3)	O2—C3—C4—O1	−0.1 (5)
C8—S1—C10—N1	−0.1 (3)	O2—C3—C4—C5	179.1 (3)
C11—O2—C3—C2	−6.2 (5)	C2—C3—C4—O1	180.0 (3)
C11—O2—C3—C4	173.9 (3)	C2—C3—C4—C5	−0.9 (5)
C10—N1—C9—O3	179.8 (3)	O1—C4—C5—C6	−179.1 (3)
C10—N1—C9—C8	0.4 (4)	C3—C4—C5—C6	1.8 (5)
C9—N1—C10—S1	−0.2 (4)	C4—C5—C6—C1	−1.4 (5)
C9—N1—C10—S2	−179.8 (3)	C1—C7—C8—S1	1.4 (6)
C6—C1—C2—C3	0.7 (5)	C1—C7—C8—C9	−179.5 (4)
C7—C1—C2—C3	−179.7 (3)	S1—C8—C9—O3	−179.8 (3)
C2—C1—C6—C5	0.2 (5)	S1—C8—C9—N1	−0.4 (3)
C7—C1—C6—C5	−179.4 (3)	C7—C8—C9—O3	1.0 (5)
C2—C1—C7—C8	3.1 (6)	C7—C8—C9—N1	−179.6 (3)
C6—C1—C7—C8	−177.4 (4)		

### Hydrogen-bond geometry (Å, °)

**Table:**

*D*—H···*A*	*D*—H	H···*A*	*D*···*A*	*D*—H···*A*
O1—H1···O2	0.88 (3)	2.21 (3)	2.641 (3)	109 (3)
C2—H2···S1	0.93	2.66	3.349 (3)	132
O1—H1···O4A^i^	0.88 (3)	1.85 (3)	2.622 (7)	145 (3)
N1—H1N···O1^ii^	0.86	2.05	2.899 (3)	169
O4A—H4A···O3^iii^	0.96 (8)	1.79 (8)	2.744 (7)	173 (7)
C12A—H12A···O3^iv^	0.96	2.37	3.150 (5)	139

Symmetry codes: (i) *x*, *y*, *z*+1; (ii) −*x*+1/2, *y*−1/2, *z*−3/2; (iii) −*x*+1/2, *y*+1/2, *z*+1/2; (iv) *x*+1/2, −*y*+1/2, *z*.
